# [μ-Bis(diphenyl­arsan­yl)methane-1:2κ^2^ *As*:*As*’]nona­carbonyl-1κ^3^ *C*,2κ^3^ *C*,3κ^3^ *C*-[tris­(biphenyl-4-yl)arsane-3κ*As*]-*triangulo*-triruthenium(0)

**DOI:** 10.1107/S1600536811000237

**Published:** 2011-01-15

**Authors:** Omar bin Shawkataly, Imthyaz Ahmed Khan, Siti Syaida Sirat, Chin Sing Yeap, Hoong-Kun Fun

**Affiliations:** a Chemical Sciences Programme, School of Distance Education, Universiti Sains Malaysia, 11800 USM, Penang, Malaysia; b X-ray Crystallography Unit, School of Physics, Universiti Sains Malaysia, 11800 USM, Penang, Malaysia

## Abstract

In the title *triangulo*-triruthenium compound, [Ru_3_(C_25_H_22_As_2_)(C_36_H_27_As)(CO)_9_], the bis­(diphenyl­arsan­yl)methane ligand bridges an Ru—Ru bond and the monodentate arsine ligand bonds to the third Ru atom. Both arsine ligands are equatorial with respect to the Ru_3_ triangle. In addition, each Ru atom carries one equatorial and two axial terminal carbonyl ligands. The phenyl rings of biphenyl are twisted from each other by dihedral angles of 50.5 (2), 44.5 (2) and 27.8 (2)°. The arsine-substituted phenyl rings make dihedral angles of 61.56 (18), 89.36 (18) and 83.27 (18)° with each other. The dihedral angles between the two benzene rings are 87.5 (2) and 81.95 (19)° for the two diphenyl­arsanyl groups. In the crystal, mol­ecules are linked into dimers by inter­molecular C—H⋯O hydrogen bonds. Weak inter­molecular C—H⋯π and π–π [centroid–centroid distance = 3.601 (3) Å] inter­actions stabilize the crystal structure.

## Related literature

For general background to *triangulo*-triruthenium derivatives, see: Bruce *et al.* (1985 ▶, 1988*a* ▶,*b* ▶). For related structures, see: Shawkataly *et al.* (1998 ▶, 2004 ▶, 2010 ▶, 2011 ▶). For the synthesis of Ru_3_(CO)_10_(μ-Ph_2_AsCH_2_AsPh_2_), see: Bruce *et al.* (1983 ▶). For the stability of the temperature controller used in the data collection, see: Cosier & Glazer (1986 ▶).
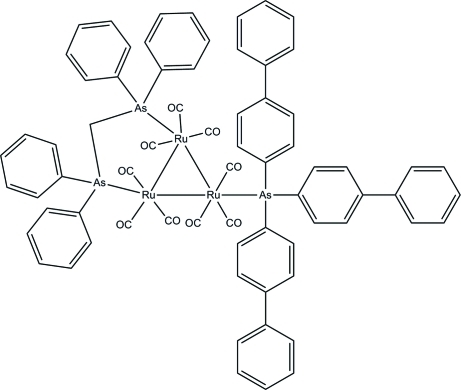


## Experimental

### 

#### Crystal data

[Ru_3_(C_25_H_22_As_2_)(C_36_H_27_As)(CO)_9_]
*M* *_r_* = 1562.06
Triclinic, 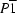

*a* = 10.906 (4) Å
*b* = 12.706 (4) Å
*c* = 22.467 (8) Å
α = 81.249 (7)°
β = 82.529 (7)°
γ = 79.301 (7)°
*V* = 3006.8 (18) Å^3^
*Z* = 2
Mo *K*α radiation
μ = 2.44 mm^−1^
*T* = 100 K
0.47 × 0.28 × 0.04 mm

#### Data collection

Bruker APEXII DUO CCD area-detector diffractometer
Absorption correction: multi-scan (*SADABS*; Bruker, 2009 ▶) *T*_min_ = 0.396, *T*_max_ = 0.900
60960 measured reflections
17294 independent reflections
13951 reflections with *I* > 2σ(*I*)
*R*_int_ = 0.035

#### Refinement

*R*[*F* ^2^ > 2σ(*F* ^2^)] = 0.037
*wR*(*F* ^2^) = 0.125
*S* = 1.11
17294 reflections
766 parameters
H-atom parameters constrained
Δρ_max_ = 1.42 e Å^−3^
Δρ_min_ = −0.69 e Å^−3^

### 

Data collection: *APEX2* (Bruker, 2009 ▶); cell refinement: *SAINT* (Bruker, 2009 ▶); data reduction: *SAINT*; program(s) used to solve structure: *SHELXTL* (Sheldrick, 2008 ▶); program(s) used to refine structure: *SHELXTL*; molecular graphics: *SHELXTL*; software used to prepare material for publication: *SHELXTL* and *PLATON* (Spek, 2009 ▶).

## Supplementary Material

Crystal structure: contains datablocks global, I. DOI: 10.1107/S1600536811000237/ng5094sup1.cif

Structure factors: contains datablocks I. DOI: 10.1107/S1600536811000237/ng5094Isup2.hkl

Additional supplementary materials:  crystallographic information; 3D view; checkCIF report

## Figures and Tables

**Table 1:** Hydrogen-bond geometry (Å, °) *Cg*1 is the centroid of the C26–C31 benzene ring.

*D*—H⋯*A*	*D*—H	H⋯*A*	*D*⋯*A*	*D*—H⋯*A*
C46—H46*A*⋯O9^i^	0.93	2.56	3.251 (6)	131
C24—H24*A*⋯*Cg*1^ii^	0.93	2.95	3.625 (5)	130

Symmetry codes: (i) 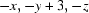; (ii) 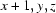.

**Fig. 1.**
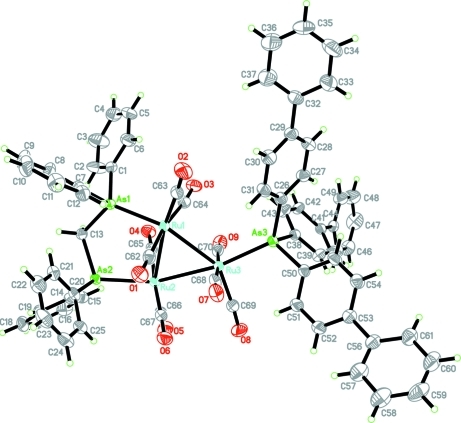
The molecular structure of the title compound with 50% probability ellipsoids for non-H atoms.

**Fig. 2.**
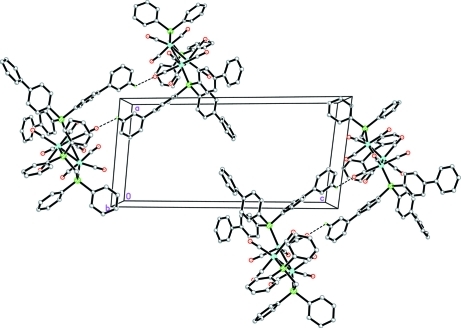
The crystal packing of the title compound, viewed down the b axis, showing the molecules are linked into dimers. Hydrogen atoms not involved in the hydrogen-bonding (dashed lines) have been omitted for clarity.

## supplementary crystallographic information

### Comment

A large number of substituted derivatives, Ru_3_(CO)_12-n_*L*_n_ (*L* = group 15 ligand) have been reported (Bruce *et al.*, 1985, 1988*a*,*b*). As part of our study on the substitution of transition metal-carbonyl clusters with mixed-ligand complexes, we have published several structures of *triangulo*-triruthenium-carbonyl clusters containing mixed P/As and P/Sb ligands (Shawkataly *et al.*, 1998, 2004, 2010). Herein we report the synthesis and structure of the title compound.

The bis(diphenylarsanyl)methane ligand bridges the Ru1–Ru2 bond and the monodentate arsine ligand bonds to the Ru3 atom. Both arsine ligands are equatorial with respect to the Ru_3_ triangle. Additionally, each Ru atom carries one equatorial and two axial terminal carbonyl ligands (Fig 1). The phenyl rings of biphenyl (C26–C31/C32–C37, C38–C43/C44–C49 and C50–C55/C56–C61) make dihedral angles of 50.5 (2), 44.5 (2) and 27.8 (2)° from each other respectively. These angles are more twisted from each other compare to the reported monodentate arsine ligand (Shawkataly *et al.*, 2011). The arsine-substituted phenyl rings make dihedral angles (C26–C31/C38–C43, C26–C31/C50–C55 and C38–C43/C50–C55) of 61.56 (18), 89.36 (18) and 83.27 (18)° with each other respectively. The dihedral angles between the two benzene rings (C1–C6/C7–C12 and C14–C19/C20–C25) are 87.5 (2) and 81.95 (19)° for the two diphenylarsanyl groups respectively.

In the crystal packing, the molecules are linked into dimers by intermolecular C46—H46A···O9 hydrogen bonds (Fig. 2, Table 1). Weak intermolecular C—H···π (Table 1) and *Cg*2···*Cg*2 interactions stabilize the crystal structure. *Cg*2···*Cg*2^iii^ = 3.601 (3) Å; *Cg*2 is centroid of C14–C19; (iii) 2 - *x*,2 - *y*,-*z*.

### Experimental

All manipulations were performed under a dry oxygen-free nitrogen atmosphere using standard Schlenk techniques. All solvents were dried over sodium and distilled from sodium benzophenone ketyl under dry oxygen free nitrogen. Tri([1,1'-biphenyl]-4-yl)arsine is prepared by the reaction of AsCl_3_ with biphenyl magnesiumbromide in THF and Ru_3_(CO)_10_(µ-Ph_2_AsCH_2_AsPh_2_) (Bruce *et al.*, 1983) was prepared by reported procedure. The title compound was obtained by refluxing equimolar quantities of Ru_3_(CO)_10_(µ-Ph_2_AsCH_2_AsPh_2_) and tri([1,1'-biphenyl]-4-yl)arsine in hexane under nitrogen atmosphere. Crystals suitable for X-ray diffraction were grown by slow solvent / solvent diffusion of CH_3_OH into CH_2_Cl_2_.

### Refinement

All hydrogen atoms were positioned geometrically and refined using a riding model with C—H = 0.93 or 0.97 Å and *U*_iso_(H) = 1.2*U*_eq_(C). The maximum and minimum residual electron density peaks of 1.42 and -0.69 e Å^-3^, respectively, were located 0.79 and 1.41 Å from the Ru3 atom.

### Crystal data

**Table:**

[Ru_3_(C_25_H_22_As_2_)(C_36_H_27_As)(CO)_9_]	*Z* = 2
*M**_r_* = 1562.06	*F*(000) = 1544
Triclinic, *P*1	*D*_x_ = 1.725 Mg m^−^^3^
Hall symbol: -P 1	Mo *K*α radiation, λ = 0.71073 Å
*a* = 10.906 (4) Å	Cell parameters from 9876 reflections
*b* = 12.706 (4) Å	θ = 2.3–30.5°
*c* = 22.467 (8) Å	µ = 2.44 mm^−^^1^
α = 81.249 (7)°	*T* = 100 K
β = 82.529 (7)°	Plate, brown
γ = 79.301 (7)°	0.47 × 0.28 × 0.04 mm
*V* = 3006.8 (18) Å^3^	

### Data collection

**Table:**

Bruker APEXII DUO CCD area-detector diffractometer	17294 independent reflections
Radiation source: fine-focus sealed tube	13951 reflections with *I* > 2σ(*I*)
graphite	*R*_int_ = 0.035
φ and ω scans	θ_max_ = 30.0°, θ_min_ = 1.7°
Absorption correction: multi-scan (*SADABS*; Bruker, 2009)	*h* = −15→15
*T*_min_ = 0.396, *T*_max_ = 0.900	*k* = −17→17
60960 measured reflections	*l* = −31→31

### Refinement

**Table:**

Refinement on *F*^2^	Primary atom site location: structure-invariant direct methods
Least-squares matrix: full	Secondary atom site location: difference Fourier map
*R*[*F*^2^ > 2σ(*F*^2^)] = 0.037	Hydrogen site location: inferred from neighbouring sites
*wR*(*F*^2^) = 0.125	H-atom parameters constrained
*S* = 1.11	*w* = 1/[σ^2^(*F*_o_^2^) + (0.0711*P*)^2^ + 2.5556*P*] where *P* = (*F*_o_^2^ + 2*F*_c_^2^)/3
17294 reflections	(Δ/σ)_max_ = 0.002
766 parameters	Δρ_max_ = 1.42 e Å^−^^3^
0 restraints	Δρ_min_ = −0.69 e Å^−^^3^

### Special details

**Table:**

Experimental. The crystal was placed in the cold stream of an Oxford Cryosystems Cobra open-flow nitrogen cryostat (Cosier & Glazer, 1986) operating at 100.0 (1) K.
Geometry. All e.s.d.'s (except the e.s.d. in the dihedral angle between two l.s. planes) are estimated using the full covariance matrix. The cell e.s.d.'s are taken into account individually in the estimation of e.s.d.'s in distances, angles and torsion angles; correlations between e.s.d.'s in cell parameters are only used when they are defined by crystal symmetry. An approximate (isotropic) treatment of cell e.s.d.'s is used for estimating e.s.d.'s involving l.s. planes.
Refinement. Refinement of *F*^2^ against ALL reflections. The weighted *R*-factor *wR* and goodness of fit *S* are based on *F*^2^, conventional *R*-factors *R* are based on *F*, with *F* set to zero for negative *F*^2^. The threshold expression of *F*^2^ > σ(*F*^2^) is used only for calculating *R*-factors(gt) *etc*. and is not relevant to the choice of reflections for refinement. *R*-factors based on *F*^2^ are statistically about twice as large as those based on *F*, and *R*- factors based on ALL data will be even larger.

### Fractional atomic coordinates and isotropic or equivalent isotropic displacement parameters (Å^2^)

**Table:**

	*x*	*y*	*z*	*U*_iso_*/*U*_eq_	
Ru1	0.44108 (2)	0.92843 (2)	0.253548 (12)	0.02048 (6)	
Ru2	0.58866 (2)	1.04717 (2)	0.162270 (12)	0.01983 (6)	
Ru3	0.38188 (2)	1.15999 (2)	0.228422 (12)	0.02075 (6)	
As1	0.52503 (3)	0.75926 (3)	0.213296 (15)	0.02132 (8)	
As2	0.74808 (3)	0.88704 (3)	0.152447 (15)	0.02072 (8)	
As3	0.17838 (3)	1.23444 (3)	0.280517 (15)	0.02213 (8)	
O1	0.6586 (3)	0.9230 (2)	0.32959 (13)	0.0382 (6)	
O2	0.2911 (3)	0.8313 (3)	0.36551 (14)	0.0465 (8)	
O3	0.2142 (3)	0.9602 (2)	0.18087 (13)	0.0366 (6)	
O4	0.4396 (3)	0.9664 (2)	0.07621 (13)	0.0350 (6)	
O5	0.6568 (3)	1.2174 (2)	0.05907 (15)	0.0463 (8)	
O6	0.7302 (3)	1.1421 (2)	0.24529 (14)	0.0388 (6)	
O7	0.5028 (3)	1.1435 (3)	0.34625 (13)	0.0397 (7)	
O8	0.4739 (3)	1.3675 (2)	0.17452 (15)	0.0472 (8)	
O9	0.2656 (3)	1.1786 (2)	0.10916 (13)	0.0378 (6)	
C1	0.4086 (3)	0.7024 (3)	0.17372 (17)	0.0268 (7)	
C2	0.4295 (5)	0.6828 (4)	0.1139 (2)	0.0499 (12)	
H2A	0.5020	0.6982	0.0901	0.060*	
C3	0.3410 (5)	0.6398 (5)	0.0895 (2)	0.0567 (14)	
H3A	0.3552	0.6266	0.0493	0.068*	
C4	0.2339 (4)	0.6168 (4)	0.1237 (2)	0.0466 (11)	
H4A	0.1776	0.5849	0.1077	0.056*	
C5	0.2104 (4)	0.6412 (4)	0.1816 (2)	0.0449 (10)	
H5A	0.1356	0.6292	0.2044	0.054*	
C6	0.2976 (4)	0.6839 (4)	0.2071 (2)	0.0400 (9)	
H6A	0.2806	0.7000	0.2467	0.048*	
C7	0.5880 (3)	0.6296 (3)	0.26612 (16)	0.0277 (7)	
C8	0.6187 (4)	0.5314 (3)	0.24233 (19)	0.0366 (9)	
H8A	0.6033	0.5275	0.2030	0.044*	
C9	0.6727 (5)	0.4394 (4)	0.2783 (2)	0.0502 (12)	
H9A	0.6923	0.3735	0.2631	0.060*	
C10	0.6972 (5)	0.4458 (4)	0.3358 (2)	0.0503 (12)	
H10A	0.7359	0.3845	0.3588	0.060*	
C11	0.6650 (4)	0.5422 (4)	0.3601 (2)	0.0447 (10)	
H11A	0.6803	0.5451	0.3995	0.054*	
C12	0.6096 (4)	0.6349 (3)	0.32528 (18)	0.0347 (8)	
H12A	0.5873	0.6999	0.3414	0.042*	
C13	0.6673 (3)	0.7630 (3)	0.14990 (15)	0.0237 (6)	
H13A	0.7276	0.6969	0.1559	0.028*	
H13B	0.6377	0.7679	0.1105	0.028*	
C14	0.8584 (3)	0.8817 (3)	0.07756 (15)	0.0239 (6)	
C15	0.8105 (4)	0.9325 (3)	0.02406 (17)	0.0310 (7)	
H15A	0.7281	0.9688	0.0250	0.037*	
C16	0.8856 (4)	0.9291 (3)	−0.03056 (18)	0.0371 (9)	
H16A	0.8530	0.9622	−0.0663	0.045*	
C17	1.0080 (4)	0.8770 (3)	−0.03219 (18)	0.0377 (9)	
H17A	1.0588	0.8767	−0.0688	0.045*	
C18	1.0558 (4)	0.8247 (4)	0.0211 (2)	0.0398 (9)	
H18A	1.1378	0.7876	0.0198	0.048*	
C19	0.9814 (4)	0.8277 (3)	0.07604 (18)	0.0321 (8)	
H19A	1.0139	0.7937	0.1117	0.038*	
C20	0.8598 (3)	0.8366 (3)	0.21505 (16)	0.0256 (7)	
C21	0.8693 (3)	0.7338 (3)	0.24714 (17)	0.0309 (8)	
H21A	0.8235	0.6851	0.2374	0.037*	
C22	0.9464 (4)	0.7020 (4)	0.2938 (2)	0.0410 (10)	
H22A	0.9511	0.6330	0.3153	0.049*	
C23	1.0157 (4)	0.7734 (4)	0.30801 (19)	0.0432 (10)	
H23A	1.0677	0.7520	0.3389	0.052*	
C24	1.0083 (4)	0.8766 (4)	0.27672 (19)	0.0373 (9)	
H24A	1.0548	0.9247	0.2866	0.045*	
C25	0.9305 (3)	0.9079 (3)	0.23013 (17)	0.0307 (7)	
H25A	0.9258	0.9771	0.2089	0.037*	
C26	0.0669 (3)	1.1485 (3)	0.33440 (15)	0.0248 (6)	
C27	−0.0622 (3)	1.1680 (3)	0.33075 (17)	0.0299 (7)	
H27A	−0.0972	1.2234	0.3028	0.036*	
C28	−0.1389 (3)	1.1042 (3)	0.36908 (17)	0.0316 (8)	
H28A	−0.2250	1.1181	0.3666	0.038*	
C29	−0.0885 (4)	1.0199 (3)	0.41117 (17)	0.0299 (7)	
C30	0.0399 (4)	1.0030 (3)	0.41462 (17)	0.0334 (8)	
H30A	0.0754	0.9479	0.4427	0.040*	
C31	0.1174 (3)	1.0674 (3)	0.37662 (17)	0.0307 (7)	
H31A	0.2032	1.0552	0.3800	0.037*	
C32	−0.1709 (4)	0.9485 (3)	0.44922 (17)	0.0324 (8)	
C33	−0.2813 (5)	0.9903 (4)	0.4816 (3)	0.0561 (13)	
H33A	−0.3067	1.0647	0.4792	0.067*	
C34	−0.3548 (5)	0.9212 (5)	0.5179 (3)	0.0611 (15)	
H34A	−0.4274	0.9508	0.5403	0.073*	
C35	−0.3238 (5)	0.8134 (4)	0.5214 (2)	0.0501 (12)	
H35A	−0.3732	0.7682	0.5459	0.060*	
C36	−0.2184 (6)	0.7728 (5)	0.4880 (3)	0.0623 (15)	
H36A	−0.1971	0.6984	0.4883	0.075*	
C37	−0.1404 (5)	0.8397 (4)	0.4530 (2)	0.0526 (12)	
H37A	−0.0664	0.8089	0.4321	0.063*	
C38	0.0643 (3)	1.3146 (3)	0.22350 (15)	0.0230 (6)	
C39	0.0431 (4)	1.4272 (3)	0.21494 (17)	0.0304 (7)	
H39A	0.0771	1.4646	0.2394	0.037*	
C40	−0.0284 (4)	1.4832 (3)	0.16998 (18)	0.0310 (8)	
H40A	−0.0401	1.5582	0.1640	0.037*	
C41	−0.0831 (3)	1.4297 (3)	0.13365 (15)	0.0239 (6)	
C42	−0.0652 (3)	1.3168 (3)	0.14421 (17)	0.0289 (7)	
H42A	−0.1038	1.2794	0.1215	0.035*	
C43	0.0087 (4)	1.2601 (3)	0.18766 (18)	0.0305 (8)	
H43A	0.0216	1.1850	0.1932	0.037*	
C44	−0.1594 (3)	1.4909 (3)	0.08523 (16)	0.0266 (7)	
C45	−0.1195 (4)	1.5767 (3)	0.04707 (18)	0.0338 (8)	
H45A	−0.0443	1.5975	0.0518	0.041*	
C46	−0.1915 (5)	1.6325 (4)	0.0014 (2)	0.0428 (10)	
H46A	−0.1636	1.6897	−0.0244	0.051*	
C47	−0.3028 (5)	1.6033 (4)	−0.0056 (2)	0.0534 (13)	
H47A	−0.3506	1.6404	−0.0360	0.064*	
C48	−0.3434 (5)	1.5187 (4)	0.0328 (3)	0.0533 (13)	
H48A	−0.4197	1.4996	0.0284	0.064*	
C49	−0.2732 (4)	1.4616 (3)	0.0778 (2)	0.0391 (9)	
H49A	−0.3015	1.4039	0.1031	0.047*	
C50	0.1902 (3)	1.3444 (3)	0.33046 (15)	0.0244 (6)	
C51	0.2992 (4)	1.3906 (3)	0.32320 (18)	0.0342 (8)	
H51A	0.3643	1.3696	0.2941	0.041*	
C52	0.3108 (4)	1.4671 (3)	0.35882 (19)	0.0358 (8)	
H52A	0.3833	1.4976	0.3529	0.043*	
C53	0.2168 (4)	1.4990 (3)	0.40307 (16)	0.0291 (7)	
C54	0.1066 (4)	1.4555 (3)	0.40865 (18)	0.0353 (8)	
H54A	0.0407	1.4779	0.4371	0.042*	
C55	0.0933 (3)	1.3797 (3)	0.37274 (17)	0.0316 (8)	
H55A	0.0188	1.3523	0.3771	0.038*	
C56	0.2369 (4)	1.5752 (3)	0.44435 (17)	0.0329 (8)	
C57	0.3567 (5)	1.5793 (4)	0.4567 (2)	0.0455 (10)	
H57A	0.4246	1.5339	0.4395	0.055*	
C58	0.3779 (6)	1.6496 (4)	0.4943 (2)	0.0534 (12)	
H58A	0.4590	1.6527	0.5016	0.064*	
C59	0.2764 (6)	1.7149 (4)	0.5207 (2)	0.0539 (13)	
H59A	0.2890	1.7613	0.5468	0.065*	
C60	0.1583 (5)	1.7119 (4)	0.5088 (2)	0.0499 (12)	
H60A	0.0907	1.7568	0.5266	0.060*	
C61	0.1370 (5)	1.6429 (4)	0.47056 (19)	0.0427 (10)	
H61A	0.0557	1.6421	0.4625	0.051*	
C62	0.5804 (3)	0.9277 (3)	0.29955 (16)	0.0286 (7)	
C63	0.3458 (3)	0.8713 (3)	0.32332 (16)	0.0284 (7)	
C64	0.3000 (3)	0.9552 (3)	0.20652 (17)	0.0286 (7)	
C65	0.4889 (3)	0.9957 (3)	0.11083 (16)	0.0258 (7)	
C66	0.6345 (3)	1.1527 (3)	0.09834 (17)	0.0285 (7)	
C67	0.6740 (3)	1.1038 (3)	0.21704 (17)	0.0275 (7)	
C68	0.4604 (3)	1.1420 (3)	0.30290 (17)	0.0309 (8)	
C69	0.4351 (4)	1.2911 (3)	0.19623 (17)	0.0307 (7)	
C70	0.3104 (3)	1.1650 (3)	0.15364 (17)	0.0273 (7)	

### Atomic displacement parameters (Å^2^)

**Table:**

	*U*^11^	*U*^22^	*U*^33^	*U*^12^	*U*^13^	*U*^23^
Ru1	0.01976 (12)	0.01985 (12)	0.02011 (13)	−0.00340 (9)	0.00132 (9)	−0.00005 (9)
Ru2	0.01677 (12)	0.01950 (12)	0.02216 (13)	−0.00391 (8)	0.00082 (9)	−0.00080 (9)
Ru3	0.01889 (12)	0.01950 (12)	0.02233 (13)	−0.00209 (9)	0.00005 (9)	−0.00126 (9)
As1	0.02062 (16)	0.01881 (15)	0.02345 (17)	−0.00429 (11)	−0.00019 (12)	−0.00001 (12)
As2	0.01733 (15)	0.02129 (16)	0.02243 (16)	−0.00327 (11)	0.00087 (11)	−0.00194 (12)
As3	0.02003 (16)	0.02089 (16)	0.02465 (17)	−0.00300 (11)	−0.00142 (12)	−0.00176 (13)
O1	0.0354 (15)	0.0456 (17)	0.0347 (15)	−0.0059 (12)	−0.0110 (12)	−0.0038 (13)
O2	0.0426 (17)	0.0522 (19)	0.0364 (16)	−0.0097 (14)	0.0092 (13)	0.0106 (14)
O3	0.0291 (14)	0.0372 (15)	0.0431 (16)	−0.0121 (11)	−0.0083 (11)	0.0078 (12)
O4	0.0311 (14)	0.0424 (16)	0.0343 (15)	−0.0107 (11)	−0.0059 (11)	−0.0066 (12)
O5	0.056 (2)	0.0318 (15)	0.0450 (18)	−0.0122 (13)	0.0121 (14)	0.0067 (13)
O6	0.0316 (15)	0.0457 (17)	0.0443 (17)	−0.0117 (12)	−0.0043 (12)	−0.0152 (13)
O7	0.0318 (14)	0.0539 (18)	0.0347 (15)	0.0011 (12)	−0.0077 (11)	−0.0166 (13)
O8	0.0529 (19)	0.0315 (15)	0.056 (2)	−0.0196 (13)	0.0132 (15)	−0.0029 (14)
O9	0.0440 (16)	0.0357 (15)	0.0319 (15)	0.0008 (12)	−0.0127 (12)	−0.0006 (12)
C1	0.0234 (16)	0.0224 (16)	0.0353 (19)	−0.0054 (12)	−0.0047 (13)	−0.0030 (14)
C2	0.051 (3)	0.073 (3)	0.036 (2)	−0.040 (2)	0.0019 (19)	−0.008 (2)
C3	0.054 (3)	0.083 (4)	0.045 (3)	−0.034 (3)	−0.010 (2)	−0.016 (3)
C4	0.036 (2)	0.044 (2)	0.067 (3)	−0.0111 (18)	−0.019 (2)	−0.015 (2)
C5	0.029 (2)	0.048 (3)	0.061 (3)	−0.0174 (17)	0.0042 (18)	−0.011 (2)
C6	0.034 (2)	0.045 (2)	0.043 (2)	−0.0143 (17)	0.0057 (17)	−0.0125 (19)
C7	0.0248 (16)	0.0247 (16)	0.0299 (18)	−0.0036 (12)	−0.0008 (13)	0.0049 (14)
C8	0.042 (2)	0.0248 (18)	0.036 (2)	0.0019 (15)	0.0050 (16)	−0.0003 (15)
C9	0.059 (3)	0.028 (2)	0.053 (3)	0.0093 (19)	0.000 (2)	0.0007 (19)
C10	0.049 (3)	0.043 (2)	0.044 (3)	0.012 (2)	−0.001 (2)	0.014 (2)
C11	0.046 (2)	0.050 (3)	0.031 (2)	−0.0034 (19)	−0.0022 (17)	0.0101 (18)
C12	0.0329 (19)	0.037 (2)	0.0297 (19)	−0.0032 (15)	0.0015 (14)	0.0031 (16)
C13	0.0237 (15)	0.0227 (15)	0.0247 (16)	−0.0045 (12)	0.0011 (12)	−0.0055 (12)
C14	0.0225 (15)	0.0242 (16)	0.0246 (16)	−0.0068 (12)	0.0052 (12)	−0.0046 (13)
C15	0.0316 (19)	0.0348 (19)	0.0291 (18)	−0.0110 (14)	−0.0010 (14)	−0.0073 (15)
C16	0.047 (2)	0.043 (2)	0.0264 (19)	−0.0182 (18)	−0.0014 (16)	−0.0081 (16)
C17	0.047 (2)	0.038 (2)	0.0292 (19)	−0.0172 (17)	0.0161 (16)	−0.0128 (16)
C18	0.034 (2)	0.040 (2)	0.041 (2)	−0.0014 (16)	0.0132 (16)	−0.0107 (18)
C19	0.0274 (18)	0.0303 (18)	0.035 (2)	−0.0027 (14)	0.0055 (14)	−0.0030 (15)
C20	0.0176 (14)	0.0336 (18)	0.0244 (16)	−0.0009 (12)	0.0013 (11)	−0.0067 (14)
C21	0.0259 (17)	0.0339 (19)	0.0300 (18)	0.0008 (14)	−0.0012 (13)	−0.0032 (15)
C22	0.036 (2)	0.045 (2)	0.037 (2)	0.0000 (17)	−0.0081 (16)	0.0065 (18)
C23	0.030 (2)	0.066 (3)	0.029 (2)	−0.0014 (19)	−0.0037 (15)	0.0001 (19)
C24	0.0249 (18)	0.055 (3)	0.035 (2)	−0.0089 (16)	−0.0029 (14)	−0.0129 (18)
C25	0.0239 (17)	0.0360 (19)	0.0316 (19)	−0.0060 (14)	0.0009 (13)	−0.0048 (15)
C26	0.0244 (16)	0.0269 (16)	0.0224 (16)	−0.0039 (12)	−0.0006 (12)	−0.0026 (13)
C27	0.0279 (17)	0.0279 (18)	0.0337 (19)	−0.0065 (13)	−0.0060 (14)	0.0009 (15)
C28	0.0247 (17)	0.039 (2)	0.0311 (19)	−0.0076 (14)	−0.0018 (13)	−0.0036 (15)
C29	0.0325 (19)	0.0311 (18)	0.0262 (17)	−0.0096 (14)	0.0020 (13)	−0.0037 (14)
C30	0.035 (2)	0.034 (2)	0.0286 (19)	−0.0043 (15)	−0.0041 (14)	0.0035 (15)
C31	0.0243 (17)	0.0347 (19)	0.0316 (19)	−0.0034 (14)	−0.0050 (13)	0.0007 (15)
C32	0.0318 (19)	0.039 (2)	0.0253 (18)	−0.0092 (15)	−0.0009 (14)	0.0003 (15)
C33	0.046 (3)	0.047 (3)	0.069 (3)	−0.009 (2)	0.012 (2)	0.000 (2)
C34	0.039 (3)	0.073 (4)	0.063 (3)	−0.012 (2)	0.012 (2)	0.005 (3)
C35	0.057 (3)	0.050 (3)	0.045 (3)	−0.027 (2)	−0.004 (2)	0.010 (2)
C36	0.074 (4)	0.047 (3)	0.058 (3)	−0.017 (3)	0.012 (3)	0.010 (2)
C37	0.065 (3)	0.046 (3)	0.041 (3)	−0.015 (2)	0.011 (2)	0.004 (2)
C38	0.0220 (15)	0.0214 (15)	0.0241 (16)	−0.0032 (11)	−0.0004 (11)	−0.0005 (12)
C39	0.0366 (19)	0.0242 (17)	0.0325 (19)	−0.0063 (14)	−0.0086 (15)	−0.0044 (14)
C40	0.0355 (19)	0.0180 (15)	0.040 (2)	−0.0062 (13)	−0.0075 (15)	−0.0015 (14)
C41	0.0229 (15)	0.0229 (16)	0.0249 (16)	−0.0037 (12)	−0.0014 (12)	−0.0007 (12)
C42	0.0310 (18)	0.0234 (16)	0.0343 (19)	−0.0046 (13)	−0.0088 (14)	−0.0053 (14)
C43	0.037 (2)	0.0173 (15)	0.038 (2)	−0.0025 (13)	−0.0107 (15)	−0.0026 (14)
C44	0.0270 (17)	0.0237 (16)	0.0278 (17)	−0.0009 (12)	−0.0038 (13)	−0.0031 (13)
C45	0.037 (2)	0.0290 (18)	0.034 (2)	−0.0054 (15)	−0.0045 (15)	0.0003 (15)
C46	0.055 (3)	0.034 (2)	0.036 (2)	−0.0017 (18)	−0.0108 (19)	0.0039 (17)
C47	0.065 (3)	0.050 (3)	0.046 (3)	0.000 (2)	−0.032 (2)	0.002 (2)
C48	0.049 (3)	0.053 (3)	0.065 (3)	−0.013 (2)	−0.030 (2)	0.000 (2)
C49	0.035 (2)	0.034 (2)	0.050 (2)	−0.0079 (16)	−0.0153 (18)	0.0019 (18)
C50	0.0238 (16)	0.0253 (16)	0.0232 (16)	−0.0030 (12)	−0.0002 (12)	−0.0035 (13)
C51	0.0293 (19)	0.039 (2)	0.037 (2)	−0.0099 (15)	0.0053 (15)	−0.0140 (17)
C52	0.0297 (19)	0.039 (2)	0.040 (2)	−0.0118 (15)	0.0053 (15)	−0.0105 (17)
C53	0.0354 (19)	0.0286 (18)	0.0226 (16)	−0.0073 (14)	−0.0006 (13)	−0.0007 (14)
C54	0.033 (2)	0.042 (2)	0.0310 (19)	−0.0096 (16)	0.0054 (15)	−0.0104 (16)
C55	0.0253 (17)	0.039 (2)	0.0309 (19)	−0.0102 (14)	0.0048 (13)	−0.0065 (15)
C56	0.044 (2)	0.0308 (19)	0.0248 (18)	−0.0128 (15)	0.0035 (15)	−0.0036 (14)
C57	0.058 (3)	0.048 (3)	0.034 (2)	−0.010 (2)	−0.0101 (19)	−0.0077 (19)
C58	0.076 (4)	0.053 (3)	0.037 (2)	−0.015 (3)	−0.017 (2)	−0.010 (2)
C59	0.089 (4)	0.047 (3)	0.033 (2)	−0.029 (3)	−0.002 (2)	−0.011 (2)
C60	0.076 (4)	0.045 (3)	0.029 (2)	−0.017 (2)	0.014 (2)	−0.0104 (19)
C61	0.055 (3)	0.040 (2)	0.032 (2)	−0.0124 (19)	0.0085 (18)	−0.0083 (18)
C62	0.0303 (18)	0.0279 (17)	0.0250 (17)	−0.0008 (13)	0.0005 (13)	−0.0031 (14)
C63	0.0274 (17)	0.0266 (17)	0.0275 (17)	−0.0016 (13)	0.0008 (13)	0.0018 (14)
C64	0.0293 (18)	0.0234 (16)	0.0296 (18)	−0.0051 (13)	0.0005 (13)	0.0047 (13)
C65	0.0203 (15)	0.0270 (17)	0.0284 (17)	−0.0029 (12)	−0.0010 (12)	−0.0013 (13)
C66	0.0246 (17)	0.0257 (17)	0.0334 (19)	−0.0062 (13)	0.0048 (13)	−0.0021 (14)
C67	0.0220 (16)	0.0275 (17)	0.0329 (18)	−0.0044 (12)	0.0012 (13)	−0.0069 (14)
C68	0.0223 (16)	0.037 (2)	0.0309 (19)	0.0013 (14)	0.0011 (13)	−0.0070 (15)
C69	0.0291 (18)	0.0300 (18)	0.0323 (19)	−0.0064 (14)	0.0034 (14)	−0.0060 (15)
C70	0.0294 (17)	0.0198 (15)	0.0304 (18)	−0.0008 (12)	−0.0033 (13)	0.0002 (13)

### Geometric parameters (Å, °)

**Table:**

Ru1—C63	1.891 (4)	C22—H22A	0.9300
Ru1—C64	1.928 (4)	C23—C24	1.384 (7)
Ru1—C62	1.943 (4)	C23—H23A	0.9300
Ru1—As1	2.4335 (8)	C24—C25	1.397 (5)
Ru1—Ru2	2.8590 (7)	C24—H24A	0.9300
Ru1—Ru3	2.8767 (10)	C25—H25A	0.9300
Ru2—C66	1.894 (4)	C26—C31	1.376 (5)
Ru2—C67	1.927 (4)	C26—C27	1.395 (5)
Ru2—C65	1.941 (4)	C27—C28	1.396 (5)
Ru2—As2	2.4361 (8)	C27—H27A	0.9300
Ru2—Ru3	2.8153 (7)	C28—C29	1.397 (5)
Ru3—C69	1.876 (4)	C28—H28A	0.9300
Ru3—C70	1.930 (4)	C29—C30	1.388 (5)
Ru3—C68	1.942 (4)	C29—C32	1.493 (5)
Ru3—As3	2.4641 (8)	C30—C31	1.403 (5)
As1—C1	1.938 (4)	C30—H30A	0.9300
As1—C7	1.947 (3)	C31—H31A	0.9300
As1—C13	1.967 (3)	C32—C37	1.353 (6)
As2—C20	1.938 (3)	C32—C33	1.383 (6)
As2—C14	1.940 (3)	C33—C34	1.397 (7)
As2—C13	1.954 (3)	C33—H33A	0.9300
As3—C38	1.943 (3)	C34—C35	1.340 (8)
As3—C50	1.952 (4)	C34—H34A	0.9300
As3—C26	1.960 (3)	C35—C36	1.350 (8)
O1—C62	1.143 (5)	C35—H35A	0.9300
O2—C63	1.154 (4)	C36—C37	1.398 (7)
O3—C64	1.149 (5)	C36—H36A	0.9300
O4—C65	1.142 (4)	C37—H37A	0.9300
O5—C66	1.145 (4)	C38—C39	1.393 (5)
O6—C67	1.149 (4)	C38—C43	1.396 (5)
O7—C68	1.135 (5)	C39—C40	1.384 (5)
O8—C69	1.146 (5)	C39—H39A	0.9300
O9—C70	1.145 (4)	C40—C41	1.389 (5)
C1—C6	1.378 (5)	C40—H40A	0.9300
C1—C2	1.387 (6)	C41—C42	1.399 (5)
C2—C3	1.399 (6)	C41—C44	1.489 (5)
C2—H2A	0.9300	C42—C43	1.377 (5)
C3—C4	1.366 (7)	C42—H42A	0.9300
C3—H3A	0.9300	C43—H43A	0.9300
C4—C5	1.364 (7)	C44—C45	1.381 (5)
C4—H4A	0.9300	C44—C49	1.396 (5)
C5—C6	1.398 (6)	C45—C46	1.398 (6)
C5—H5A	0.9300	C45—H45A	0.9300
C6—H6A	0.9300	C46—C47	1.367 (7)
C7—C12	1.393 (6)	C46—H46A	0.9300
C7—C8	1.399 (5)	C47—C48	1.375 (7)
C8—C9	1.395 (6)	C47—H47A	0.9300
C8—H8A	0.9300	C48—C49	1.381 (6)
C9—C10	1.370 (7)	C48—H48A	0.9300
C9—H9A	0.9300	C49—H49A	0.9300
C10—C11	1.385 (7)	C50—C55	1.383 (5)
C10—H10A	0.9300	C50—C51	1.401 (5)
C11—C12	1.394 (6)	C51—C52	1.381 (6)
C11—H11A	0.9300	C51—H51A	0.9300
C12—H12A	0.9300	C52—C53	1.381 (5)
C13—H13A	0.9700	C52—H52A	0.9300
C13—H13B	0.9700	C53—C54	1.398 (5)
C14—C19	1.387 (5)	C53—C56	1.500 (5)
C14—C15	1.392 (5)	C54—C55	1.385 (6)
C15—C16	1.386 (5)	C54—H54A	0.9300
C15—H15A	0.9300	C55—H55A	0.9300
C16—C17	1.374 (6)	C56—C57	1.383 (6)
C16—H16A	0.9300	C56—C61	1.383 (6)
C17—C18	1.391 (6)	C57—C58	1.386 (7)
C17—H17A	0.9300	C57—H57A	0.9300
C18—C19	1.388 (5)	C58—C59	1.379 (8)
C18—H18A	0.9300	C58—H58A	0.9300
C19—H19A	0.9300	C59—C60	1.358 (8)
C20—C21	1.386 (5)	C59—H59A	0.9300
C20—C25	1.397 (5)	C60—C61	1.386 (7)
C21—C22	1.395 (5)	C60—H60A	0.9300
C21—H21A	0.9300	C61—H61A	0.9300
C22—C23	1.378 (7)		
			
C63—Ru1—C64	92.14 (15)	C23—C22—H22A	120.1
C63—Ru1—C62	91.57 (16)	C21—C22—H22A	120.1
C64—Ru1—C62	170.39 (15)	C22—C23—C24	120.5 (4)
C63—Ru1—As1	97.75 (11)	C22—C23—H23A	119.7
C64—Ru1—As1	91.93 (11)	C24—C23—H23A	119.7
C62—Ru1—As1	96.35 (11)	C23—C24—C25	119.4 (4)
C63—Ru1—Ru2	168.63 (12)	C23—C24—H24A	120.3
C64—Ru1—Ru2	92.96 (10)	C25—C24—H24A	120.3
C62—Ru1—Ru2	81.88 (11)	C20—C25—C24	120.8 (4)
C63—Ru1—Ru3	112.97 (11)	C20—C25—H25A	119.6
C64—Ru1—Ru3	75.28 (11)	C24—C25—H25A	119.6
C62—Ru1—Ru3	95.11 (11)	C31—C26—C27	119.5 (3)
As1—Ru1—Ru3	146.81 (2)	C31—C26—As3	119.2 (3)
Ru2—Ru1—Ru3	58.791 (16)	C27—C26—As3	121.3 (3)
C66—Ru2—C67	90.62 (16)	C26—C27—C28	120.0 (3)
C66—Ru2—C65	90.87 (16)	C26—C27—H27A	120.0
C67—Ru2—C65	174.96 (14)	C28—C27—H27A	120.0
C66—Ru2—As2	105.56 (11)	C27—C28—C29	121.1 (3)
C67—Ru2—As2	94.76 (11)	C27—C28—H28A	119.5
C65—Ru2—As2	89.47 (10)	C29—C28—H28A	119.5
C66—Ru2—Ru3	103.67 (11)	C30—C29—C28	117.9 (3)
C67—Ru2—Ru3	79.84 (11)	C30—C29—C32	121.7 (3)
C65—Ru2—Ru3	95.13 (10)	C28—C29—C32	120.3 (3)
As2—Ru2—Ru3	150.318 (16)	C29—C30—C31	121.3 (3)
C66—Ru2—Ru1	161.39 (11)	C29—C30—H30A	119.4
C67—Ru2—Ru1	96.28 (11)	C31—C30—H30A	119.4
C65—Ru2—Ru1	80.88 (10)	C26—C31—C30	120.2 (3)
Ru3—Ru2—Ru1	60.92 (2)	C26—C31—H31A	119.9
C69—Ru3—C70	87.66 (16)	C30—C31—H31A	119.9
C69—Ru3—C68	95.64 (17)	C37—C32—C33	117.3 (4)
C70—Ru3—C68	175.00 (15)	C37—C32—C29	120.9 (4)
C69—Ru3—As3	98.12 (12)	C33—C32—C29	121.7 (4)
C70—Ru3—As3	92.47 (11)	C32—C33—C34	120.3 (5)
C68—Ru3—As3	90.78 (11)	C32—C33—H33A	119.8
C69—Ru3—Ru2	90.05 (12)	C34—C33—H33A	119.8
C70—Ru3—Ru2	80.53 (11)	C35—C34—C33	121.9 (5)
C68—Ru3—Ru2	95.68 (11)	C35—C34—H34A	119.0
As3—Ru3—Ru2	169.049 (15)	C33—C34—H34A	119.0
C69—Ru3—Ru1	148.56 (12)	C34—C35—C36	117.7 (5)
C70—Ru3—Ru1	96.67 (10)	C34—C35—H35A	121.2
C68—Ru3—Ru1	78.56 (12)	C36—C35—H35A	121.2
As3—Ru3—Ru1	112.715 (16)	C35—C36—C37	121.8 (5)
Ru2—Ru3—Ru1	60.291 (14)	C35—C36—H36A	119.1
C1—As1—C7	99.53 (15)	C37—C36—H36A	119.1
C1—As1—C13	101.37 (15)	C32—C37—C36	120.9 (5)
C7—As1—C13	100.45 (15)	C32—C37—H37A	119.5
C1—As1—Ru1	115.25 (11)	C36—C37—H37A	119.5
C7—As1—Ru1	121.01 (12)	C39—C38—C43	119.0 (3)
C13—As1—Ru1	116.05 (10)	C39—C38—As3	120.6 (3)
C20—As2—C14	104.05 (15)	C43—C38—As3	120.3 (2)
C20—As2—C13	103.72 (15)	C40—C39—C38	120.0 (3)
C14—As2—C13	99.28 (14)	C40—C39—H39A	120.0
C20—As2—Ru2	119.35 (10)	C38—C39—H39A	120.0
C14—As2—Ru2	118.20 (10)	C39—C40—C41	121.5 (3)
C13—As2—Ru2	109.56 (10)	C39—C40—H40A	119.3
C38—As3—C50	101.80 (14)	C41—C40—H40A	119.3
C38—As3—C26	100.91 (14)	C40—C41—C42	118.0 (3)
C50—As3—C26	101.33 (15)	C40—C41—C44	121.0 (3)
C38—As3—Ru3	111.64 (10)	C42—C41—C44	121.0 (3)
C50—As3—Ru3	113.29 (10)	C43—C42—C41	121.0 (3)
C26—As3—Ru3	124.89 (10)	C43—C42—H42A	119.5
C6—C1—C2	119.0 (4)	C41—C42—H42A	119.5
C6—C1—As1	117.3 (3)	C42—C43—C38	120.5 (3)
C2—C1—As1	123.7 (3)	C42—C43—H43A	119.8
C1—C2—C3	119.7 (4)	C38—C43—H43A	119.8
C1—C2—H2A	120.2	C45—C44—C49	119.0 (3)
C3—C2—H2A	120.2	C45—C44—C41	121.1 (3)
C4—C3—C2	121.0 (5)	C49—C44—C41	119.9 (3)
C4—C3—H3A	119.5	C44—C45—C46	120.3 (4)
C2—C3—H3A	119.5	C44—C45—H45A	119.8
C5—C4—C3	119.3 (4)	C46—C45—H45A	119.8
C5—C4—H4A	120.4	C47—C46—C45	120.3 (4)
C3—C4—H4A	120.4	C47—C46—H46A	119.8
C4—C5—C6	120.7 (4)	C45—C46—H46A	119.8
C4—C5—H5A	119.6	C46—C47—C48	119.5 (4)
C6—C5—H5A	119.6	C46—C47—H47A	120.3
C1—C6—C5	120.2 (4)	C48—C47—H47A	120.3
C1—C6—H6A	119.9	C47—C48—C49	121.3 (4)
C5—C6—H6A	119.9	C47—C48—H48A	119.4
C12—C7—C8	120.3 (3)	C49—C48—H48A	119.4
C12—C7—As1	121.0 (3)	C48—C49—C44	119.6 (4)
C8—C7—As1	118.6 (3)	C48—C49—H49A	120.2
C9—C8—C7	119.2 (4)	C44—C49—H49A	120.2
C9—C8—H8A	120.4	C55—C50—C51	118.4 (3)
C7—C8—H8A	120.4	C55—C50—As3	122.0 (3)
C10—C9—C8	120.3 (4)	C51—C50—As3	119.6 (3)
C10—C9—H9A	119.9	C52—C51—C50	120.7 (4)
C8—C9—H9A	119.9	C52—C51—H51A	119.7
C9—C10—C11	120.9 (4)	C50—C51—H51A	119.7
C9—C10—H10A	119.5	C53—C52—C51	121.3 (4)
C11—C10—H10A	119.5	C53—C52—H52A	119.3
C10—C11—C12	119.8 (4)	C51—C52—H52A	119.3
C10—C11—H11A	120.1	C52—C53—C54	117.8 (4)
C12—C11—H11A	120.1	C52—C53—C56	119.9 (4)
C7—C12—C11	119.5 (4)	C54—C53—C56	122.3 (3)
C7—C12—H12A	120.2	C55—C54—C53	121.4 (4)
C11—C12—H12A	120.2	C55—C54—H54A	119.3
As2—C13—As1	110.23 (16)	C53—C54—H54A	119.3
As2—C13—H13A	109.6	C50—C55—C54	120.4 (4)
As1—C13—H13A	109.6	C50—C55—H55A	119.8
As2—C13—H13B	109.6	C54—C55—H55A	119.8
As1—C13—H13B	109.6	C57—C56—C61	118.6 (4)
H13A—C13—H13B	108.1	C57—C56—C53	120.2 (4)
C19—C14—C15	119.9 (3)	C61—C56—C53	121.1 (4)
C19—C14—As2	122.0 (3)	C56—C57—C58	121.4 (5)
C15—C14—As2	118.2 (3)	C56—C57—H57A	119.3
C16—C15—C14	120.0 (4)	C58—C57—H57A	119.3
C16—C15—H15A	120.0	C59—C58—C57	118.9 (5)
C14—C15—H15A	120.0	C59—C58—H58A	120.6
C17—C16—C15	120.4 (4)	C57—C58—H58A	120.6
C17—C16—H16A	119.8	C60—C59—C58	120.4 (5)
C15—C16—H16A	119.8	C60—C59—H59A	119.8
C16—C17—C18	119.8 (4)	C58—C59—H59A	119.8
C16—C17—H17A	120.1	C59—C60—C61	120.9 (5)
C18—C17—H17A	120.1	C59—C60—H60A	119.6
C19—C18—C17	120.3 (4)	C61—C60—H60A	119.6
C19—C18—H18A	119.9	C56—C61—C60	119.9 (5)
C17—C18—H18A	119.9	C56—C61—H61A	120.1
C14—C19—C18	119.7 (4)	C60—C61—H61A	120.1
C14—C19—H19A	120.2	O1—C62—Ru1	175.5 (3)
C18—C19—H19A	120.2	O2—C63—Ru1	176.6 (3)
C21—C20—C25	118.4 (3)	O3—C64—Ru1	172.8 (3)
C21—C20—As2	122.8 (3)	O4—C65—Ru2	173.4 (3)
C25—C20—As2	118.8 (3)	O5—C66—Ru2	177.0 (4)
C20—C21—C22	121.1 (4)	O6—C67—Ru2	174.0 (3)
C20—C21—H21A	119.5	O7—C68—Ru3	172.5 (4)
C22—C21—H21A	119.5	O8—C69—Ru3	175.6 (3)
C23—C22—C21	119.7 (4)	O9—C70—Ru3	173.3 (3)
			
C63—Ru1—Ru2—C66	−82.2 (7)	C13—As1—C7—C12	116.4 (3)
C64—Ru1—Ru2—C66	34.3 (4)	Ru1—As1—C7—C12	−12.8 (4)
C62—Ru1—Ru2—C66	−137.5 (4)	C1—As1—C7—C8	43.9 (3)
As1—Ru1—Ru2—C66	126.4 (4)	C13—As1—C7—C8	−59.6 (3)
Ru3—Ru1—Ru2—C66	−36.5 (4)	Ru1—As1—C7—C8	171.2 (3)
C63—Ru1—Ru2—C67	28.9 (6)	C12—C7—C8—C9	−0.9 (6)
C64—Ru1—Ru2—C67	145.48 (15)	As1—C7—C8—C9	175.2 (3)
C62—Ru1—Ru2—C67	−26.37 (15)	C7—C8—C9—C10	−1.0 (7)
As1—Ru1—Ru2—C67	−122.47 (11)	C8—C9—C10—C11	2.2 (8)
Ru3—Ru1—Ru2—C67	74.67 (11)	C9—C10—C11—C12	−1.5 (7)
C63—Ru1—Ru2—C65	−146.9 (6)	C8—C7—C12—C11	1.6 (6)
C64—Ru1—Ru2—C65	−30.32 (15)	As1—C7—C12—C11	−174.4 (3)
C62—Ru1—Ru2—C65	157.83 (15)	C10—C11—C12—C7	−0.4 (6)
As1—Ru1—Ru2—C65	61.74 (10)	C20—As2—C13—As1	83.00 (18)
Ru3—Ru1—Ru2—C65	−101.12 (10)	C14—As2—C13—As1	−169.96 (17)
C63—Ru1—Ru2—As2	123.8 (5)	Ru2—As2—C13—As1	−45.47 (17)
C64—Ru1—Ru2—As2	−119.61 (11)	C1—As1—C13—As2	145.49 (17)
C62—Ru1—Ru2—As2	68.54 (11)	C7—As1—C13—As2	−112.46 (18)
As1—Ru1—Ru2—As2	−27.553 (17)	Ru1—As1—C13—As2	19.86 (19)
Ru3—Ru1—Ru2—As2	169.586 (14)	C20—As2—C14—C19	13.3 (3)
C63—Ru1—Ru2—Ru3	−45.7 (5)	C13—As2—C14—C19	−93.5 (3)
C64—Ru1—Ru2—Ru3	70.81 (11)	Ru2—As2—C14—C19	148.3 (3)
C62—Ru1—Ru2—Ru3	−101.05 (11)	C20—As2—C14—C15	−167.8 (3)
As1—Ru1—Ru2—Ru3	162.861 (15)	C13—As2—C14—C15	85.4 (3)
C66—Ru2—Ru3—C69	−22.23 (17)	Ru2—As2—C14—C15	−32.8 (3)
C67—Ru2—Ru3—C69	65.92 (17)	C19—C14—C15—C16	0.0 (6)
C65—Ru2—Ru3—C69	−114.38 (16)	As2—C14—C15—C16	−178.9 (3)
As2—Ru2—Ru3—C69	147.63 (12)	C14—C15—C16—C17	−0.9 (6)
Ru1—Ru2—Ru3—C69	169.04 (12)	C15—C16—C17—C18	1.9 (6)
C66—Ru2—Ru3—C70	65.40 (16)	C16—C17—C18—C19	−2.0 (6)
C67—Ru2—Ru3—C70	153.55 (15)	C15—C14—C19—C18	−0.1 (6)
C65—Ru2—Ru3—C70	−26.76 (15)	As2—C14—C19—C18	178.8 (3)
As2—Ru2—Ru3—C70	−124.75 (11)	C17—C18—C19—C14	1.0 (6)
Ru1—Ru2—Ru3—C70	−103.34 (11)	C14—As2—C20—C21	−103.8 (3)
C66—Ru2—Ru3—C68	−117.90 (17)	C13—As2—C20—C21	−0.4 (3)
C67—Ru2—Ru3—C68	−29.74 (17)	Ru2—As2—C20—C21	121.8 (3)
C65—Ru2—Ru3—C68	149.95 (16)	C14—As2—C20—C25	78.6 (3)
As2—Ru2—Ru3—C68	51.96 (13)	C13—As2—C20—C25	−178.0 (3)
Ru1—Ru2—Ru3—C68	73.37 (12)	Ru2—As2—C20—C25	−55.8 (3)
C66—Ru2—Ru3—As3	116.23 (14)	C25—C20—C21—C22	0.7 (5)
C67—Ru2—Ru3—As3	−155.62 (13)	As2—C20—C21—C22	−177.0 (3)
C65—Ru2—Ru3—As3	24.07 (13)	C20—C21—C22—C23	−0.8 (6)
As2—Ru2—Ru3—As3	−73.92 (9)	C21—C22—C23—C24	0.6 (7)
Ru1—Ru2—Ru3—As3	−52.51 (8)	C22—C23—C24—C25	−0.4 (6)
C66—Ru2—Ru3—Ru1	168.74 (12)	C21—C20—C25—C24	−0.5 (5)
C67—Ru2—Ru3—Ru1	−103.11 (11)	As2—C20—C25—C24	177.3 (3)
C65—Ru2—Ru3—Ru1	76.59 (10)	C23—C24—C25—C20	0.4 (6)
As2—Ru2—Ru3—Ru1	−21.40 (3)	C38—As3—C26—C31	170.3 (3)
C63—Ru1—Ru3—C69	149.8 (3)	C50—As3—C26—C31	−85.2 (3)
C64—Ru1—Ru3—C69	−124.2 (3)	Ru3—As3—C26—C31	43.9 (3)
C62—Ru1—Ru3—C69	55.9 (3)	C38—As3—C26—C27	−9.7 (3)
As1—Ru1—Ru3—C69	−53.9 (2)	C50—As3—C26—C27	94.8 (3)
Ru2—Ru1—Ru3—C69	−21.4 (2)	Ru3—As3—C26—C27	−136.0 (3)
C63—Ru1—Ru3—C70	−113.74 (17)	C31—C26—C27—C28	−1.0 (6)
C64—Ru1—Ru3—C70	−27.72 (16)	As3—C26—C27—C28	178.9 (3)
C62—Ru1—Ru3—C70	152.38 (16)	C26—C27—C28—C29	−0.5 (6)
As1—Ru1—Ru3—C70	42.54 (11)	C27—C28—C29—C30	1.4 (6)
Ru2—Ru1—Ru3—C70	75.09 (11)	C27—C28—C29—C32	−176.4 (4)
C63—Ru1—Ru3—C68	67.78 (17)	C28—C29—C30—C31	−0.8 (6)
C64—Ru1—Ru3—C68	153.80 (16)	C32—C29—C30—C31	176.9 (4)
C62—Ru1—Ru3—C68	−26.10 (15)	C27—C26—C31—C30	1.6 (6)
As1—Ru1—Ru3—C68	−135.94 (11)	As3—C26—C31—C30	−178.4 (3)
Ru2—Ru1—Ru3—C68	−103.39 (11)	C29—C30—C31—C26	−0.7 (6)
C63—Ru1—Ru3—As3	−18.23 (13)	C30—C29—C32—C37	−48.6 (6)
C64—Ru1—Ru3—As3	67.79 (12)	C28—C29—C32—C37	129.0 (5)
C62—Ru1—Ru3—As3	−112.12 (11)	C30—C29—C32—C33	131.8 (5)
As1—Ru1—Ru3—As3	138.05 (3)	C28—C29—C32—C33	−50.6 (6)
Ru2—Ru1—Ru3—As3	170.595 (16)	C37—C32—C33—C34	1.9 (8)
C63—Ru1—Ru3—Ru2	171.18 (13)	C29—C32—C33—C34	−178.5 (5)
C64—Ru1—Ru3—Ru2	−102.80 (12)	C32—C33—C34—C35	−2.0 (9)
C62—Ru1—Ru3—Ru2	77.29 (11)	C33—C34—C35—C36	−0.4 (9)
As1—Ru1—Ru3—Ru2	−32.54 (3)	C34—C35—C36—C37	2.9 (9)
C63—Ru1—As1—C1	76.64 (17)	C33—C32—C37—C36	0.5 (8)
C64—Ru1—As1—C1	−15.78 (16)	C29—C32—C37—C36	−179.1 (5)
C62—Ru1—As1—C1	169.10 (16)	C35—C36—C37—C32	−3.0 (9)
Ru2—Ru1—As1—C1	−108.83 (12)	C50—As3—C38—C39	18.2 (3)
Ru3—Ru1—As1—C1	−81.41 (12)	C26—As3—C38—C39	122.4 (3)
C63—Ru1—As1—C7	−43.16 (17)	Ru3—As3—C38—C39	−102.9 (3)
C64—Ru1—As1—C7	−135.58 (16)	C50—As3—C38—C43	−166.0 (3)
C62—Ru1—As1—C7	49.31 (16)	C26—As3—C38—C43	−61.8 (3)
Ru2—Ru1—As1—C7	131.38 (12)	Ru3—As3—C38—C43	72.9 (3)
Ru3—Ru1—As1—C7	158.79 (12)	C43—C38—C39—C40	−2.3 (6)
C63—Ru1—As1—C13	−165.12 (16)	As3—C38—C39—C40	173.6 (3)
C64—Ru1—As1—C13	102.45 (15)	C38—C39—C40—C41	1.7 (6)
C62—Ru1—As1—C13	−72.66 (16)	C39—C40—C41—C42	0.8 (6)
Ru2—Ru1—As1—C13	9.41 (11)	C39—C40—C41—C44	−179.7 (4)
Ru3—Ru1—As1—C13	36.82 (12)	C40—C41—C42—C43	−2.6 (6)
C66—Ru2—As2—C20	114.01 (17)	C44—C41—C42—C43	177.9 (4)
C67—Ru2—As2—C20	22.03 (16)	C41—C42—C43—C38	2.0 (6)
C65—Ru2—As2—C20	−155.24 (16)	C39—C38—C43—C42	0.5 (6)
Ru3—Ru2—As2—C20	−55.76 (13)	As3—C38—C43—C42	−175.4 (3)
Ru1—Ru2—As2—C20	−74.37 (12)	C40—C41—C44—C45	44.1 (5)
C66—Ru2—As2—C14	−14.13 (17)	C42—C41—C44—C45	−136.4 (4)
C67—Ru2—As2—C14	−106.11 (16)	C40—C41—C44—C49	−136.0 (4)
C65—Ru2—As2—C14	76.63 (16)	C42—C41—C44—C49	43.5 (5)
Ru3—Ru2—As2—C14	176.10 (12)	C49—C44—C45—C46	−0.7 (6)
Ru1—Ru2—As2—C14	157.50 (12)	C41—C44—C45—C46	179.2 (4)
C66—Ru2—As2—C13	−126.76 (16)	C44—C45—C46—C47	0.7 (7)
C67—Ru2—As2—C13	141.27 (15)	C45—C46—C47—C48	0.1 (8)
C65—Ru2—As2—C13	−36.00 (15)	C46—C47—C48—C49	−0.9 (9)
Ru3—Ru2—As2—C13	63.47 (11)	C47—C48—C49—C44	0.9 (8)
Ru1—Ru2—As2—C13	44.87 (11)	C45—C44—C49—C48	−0.1 (7)
C69—Ru3—As3—C38	65.34 (16)	C41—C44—C49—C48	180.0 (4)
C70—Ru3—As3—C38	−22.65 (15)	C38—As3—C50—C55	73.7 (3)
C68—Ru3—As3—C38	161.15 (16)	C26—As3—C50—C55	−30.1 (3)
Ru2—Ru3—As3—C38	−72.59 (13)	Ru3—As3—C50—C55	−166.3 (3)
Ru1—Ru3—As3—C38	−120.94 (11)	C38—As3—C50—C51	−105.5 (3)
C69—Ru3—As3—C50	−48.88 (16)	C26—As3—C50—C51	150.7 (3)
C70—Ru3—As3—C50	−136.87 (15)	Ru3—As3—C50—C51	14.5 (3)
C68—Ru3—As3—C50	46.93 (16)	C55—C50—C51—C52	2.0 (6)
Ru2—Ru3—As3—C50	173.19 (13)	As3—C50—C51—C52	−178.7 (3)
Ru1—Ru3—As3—C50	124.84 (11)	C50—C51—C52—C53	1.0 (6)
C69—Ru3—As3—C26	−173.00 (17)	C51—C52—C53—C54	−3.2 (6)
C70—Ru3—As3—C26	99.01 (16)	C51—C52—C53—C56	174.9 (4)
C68—Ru3—As3—C26	−77.19 (17)	C52—C53—C54—C55	2.5 (6)
Ru2—Ru3—As3—C26	49.07 (15)	C56—C53—C54—C55	−175.6 (4)
Ru1—Ru3—As3—C26	0.73 (13)	C51—C50—C55—C54	−2.7 (6)
C7—As1—C1—C6	74.2 (3)	As3—C50—C55—C54	178.1 (3)
C13—As1—C1—C6	177.0 (3)	C53—C54—C55—C50	0.5 (6)
Ru1—As1—C1—C6	−56.8 (3)	C52—C53—C56—C57	−27.0 (6)
C7—As1—C1—C2	−107.8 (4)	C54—C53—C56—C57	151.1 (4)
C13—As1—C1—C2	−5.0 (4)	C52—C53—C56—C61	152.8 (4)
Ru1—As1—C1—C2	121.2 (4)	C54—C53—C56—C61	−29.2 (6)
C6—C1—C2—C3	−3.2 (7)	C61—C56—C57—C58	−0.3 (7)
As1—C1—C2—C3	178.8 (4)	C53—C56—C57—C58	179.4 (4)
C1—C2—C3—C4	0.1 (9)	C56—C57—C58—C59	1.4 (7)
C2—C3—C4—C5	3.2 (9)	C57—C58—C59—C60	−1.5 (8)
C3—C4—C5—C6	−3.3 (8)	C58—C59—C60—C61	0.6 (7)
C2—C1—C6—C5	3.0 (7)	C57—C56—C61—C60	−0.7 (6)
As1—C1—C6—C5	−178.8 (3)	C53—C56—C61—C60	179.6 (4)
C4—C5—C6—C1	0.2 (7)	C59—C60—C61—C56	0.6 (7)
C1—As1—C7—C12	−140.1 (3)		

### Hydrogen-bond geometry (Å, °)

**Table:**

Cg1 is the centroid of the C26–C31 benzene ring.

**Table:**

*D*—H···*A*	*D*—H	H···*A*	*D*···*A*	*D*—H···*A*
C46—H46A···O9^i^	0.93	2.56	3.251 (6)	131
C24—H24A···Cg1^ii^	0.93	2.95	3.625 (5)	130

Symmetry codes: (i) −*x*, −*y*+3, −*z*; (ii) *x*+1, *y*, *z*.
